# Results of a Survey Offering Clinical Insights into Speech-Language Pathology Telepractice Methods

**DOI:** 10.5195/ijt.2017.6230

**Published:** 2017-11-20

**Authors:** ELIZABETH U. GRILLO

**Affiliations:** WEST CHESTER UNIVERSITY, DEPARTMENT OF COMMUNICATION SCIENCES AND DISORDERS, WEST CHESTER, PA, USA

**Keywords:** eHealth, Telehealth, Telemedicine, Telepractice

## Abstract

A telepractice survey was administered to the American Speech-Language-Hearing Association Special Interest Group 18 Telepractice affiliates and attendees of the Waldo County General Hospital Speech-Language Pathology Telepractice Training program in Maine, USA over the summer of 2016. Sixty-seven respondents completed the survey. The survey explored demographics of clients and clinicians, costs and equipment, learning opportunities, use of the client’s environment and caregivers/e-helpers, and method adaptations in telepractice. The results of the survey provide information on the current state of telepractice methods in speech-language pathology from experienced practitioners. This information may be used to develop telepractice models and to prepare speech-language pathology graduate students in the delivery of telepractice methods.

By 2030, 72.1 million people in the USA will be 65 years or older and will represent 20% of the US population, expanding the need for speech-language pathology services while increasing costs (Administration on Aging, 2014). Not only are demographics changing, but people are also experiencing extended work days reducing the capacity to commit to in-person services (Cason & Cohn, 2014; Pickering et al., 1998). Equitable access to services continues to challenge current service delivery models as evidenced by ongoing difficulties with recruitment and retention of speech-language pathologists in rural and remote areas and by servicing bilingual populations with qualified speech-language pathologists (Cason & Cohn, 2014; Pickering et al., 1998). Furthermore, many individuals with communication disorders also have co-occurring physical disabilities that prohibit access to in-person services.

Telepractice may offer a solution by providing convenient and cost effective access to speech-language pathology (SLP) services at a distance. While the advantages of telepractice are obvious in terms of reducing costs and improving access, another benefit of telepractice is found with the provision of services to clients in their functional environments, which is considered best practices in many areas of rehabilitation (McCue, Fairman, & Pramuka, 2010) and is supported by the World Health Organization (WHO) intervention framework (WHO, 2001). Telepractice has the potential to improve client outcomes by targeting the functional environment, sustaining services, facilitating self-management, and reducing costs.

A 2002 survey conducted by the America Speech-Language-Hearing Association (ASHA) revealed that fewer than 21% of respondents had received training in telepractice methods (ASHA, 2002). Of those who were trained, 47% reported receiving on-the-job training, 44% completed continuing education courses, and 19% were trained during graduate school. According to a national survey that evaluated the current state of telepractice training in graduate programs, only 26% of the reporting universities were providing academic and clinical training in telepractice (Grogan-Johnson, Meehan, McCormick, & Miller, 2015). A more recent 2016 ASHA telepractice survey indicated that 58.5% of respondents received telepractice training by an employer, while only 6.9% of respondents had received telepractice training in graduate school (ASHA, 2016). When comparing the results of all three surveys over a 14-year period from 2002 to 2016, there appear to be differing results related to receiving telepractice training in graduate school. Comparing the 2002 and 2016 ASHA surveys, there were more clinicians trained in telepractice methods in graduate school in 2002 than in 2016 (ASHA 2002; 2016). That goes against what is expected considering that telepractice is more prevalent now and widely accepted, then it was 14 years ago. Also, the 2015 graduate school survey (Grogan-Johnson et al., 2015), reported that 26% of graduate programs were providing telepractice training; however, only 6.9% of respondents reported receiving training in graduate school in the 2016 ASHA survey (ASHA, 2016). It is anticipated that the number of graduate programs offering telepractice training will only increase as time advances. The differing results could have several explanations. One explanation may be that while 26% of graduate programs provided telepractice training, perhaps not all students received the training. A second explanation may be that the participants of the survey may have graduated from programs before telepractice training was offered. Regardless of the difference in results, it is clear that most clinicians do not receive telepractice training in graduate school. The majority of the training is occurring with employers.

The previous surveys provided information about demographics of clinicians and clients being served by telepractice, areas of service delivery, sources of training, and preparation for telepractice (ASHA, 2002, 2016; Grogan-Johnson et al., 2015). To design effective telepractice models with clients and to train SLP graduate students in telepractice methods, further information about telepractice was needed regarding costs, methodology differences between in-person and telepractice, types of learning opportunities offered, and manipulation of the client’s environment from clinicians who are currently using telepractice as a service delivery model. A survey was created and administered targeting the need for further information. This article will describe the results of that survey.

## METHODS

### DESCRIPTION OF THE SURVEY AND SAMPLING PROCEDURES

The survey consisted of seven sections, 66 questions, and was approved by West Chester University’s Institutional Review Board. The seven sections were (1) demographics, (2) licensing and licensure regulations, (3) costs and equipment, (4) synchronous and asynchronous learning opportunities, (5) use of the client’s environment and caregiver/e-helper interactions, (6) method adaptations, and (7) overall impressions of telepractice. A majority of the questions required a response from a selection of multiple choice options. Some of the questions were answered with either Yes or No. There were some open-ended response options where participants could provide comments. For the multiple choice questions, participants could select more than one response when it was appropriate to do so.

To ensure that experienced telepractice practitioners participated in the survey, the survey was sent to ASHA Special Interest Group (SIG) 18 affiliates through the Community Discussion Board as a web-based Qualtrics survey link. In addition, the same Qualtrics survey link was emailed to participants who attended the Waldo County General Hospital Speech-Language Pathology Telepractice Training program in Maine, USA. The survey was open and accepting responses over the summer of 2016. After respondents offered their informed consent to participate in the survey, they were directed to the first question of the survey. If respondents did not offer their informed consent to participate, then the survey ended. There were 67 participants; 59 SLPs and four audiologists. Sixty-two of the 67 respondents were providing telepractice services at the time of survey. The following results section will be organized by the seven overall sections within the survey.

## RESULTS

### PROFILE OF THE PARTICIPANTS BY DEMOGRAPHICS

The majority of respondents were servicing clients via telepractice from the ages of 6–17 years. Interestingly, all ages were represented from under six months of age to above 75 years. Treatment was the most common service offered across 93% of the respondents. Supervision of graduate student clinicians and clinical fellows were reported by 15% and 12% of the respondents, respectively. Half of the respondents reported consulting with other professionals about clients without the client or caregiver present and half of the respondents indicated that telepractice was being used for follow up or monitoring of previously learned skills. Half of the respondents reported using a hybrid approach (i.e., both in-person and telepractice sessions) to service clients. Fifty-six percent of respondents reported using only telepractice to service clients. Reasons given for using a hybrid approach versus telepractice only were: requirements of the state, professional judgment based on initial interview and assessment, distance, computer skills, and parent/caregiver involvement. Only 22% of the respondents have denied a client from participating in telepractice services and 37% of the respondents had recommended a switch from telepractice sessions to in-person only sessions. Reasons given were: client skills were better served via in-person, comorbidity (i.e., blindness, deafness, limited mental capacity, and severe dysphagia, etc.) or behaviors which significantly compromised the ability to participate in the virtual environment, bias of some team members, poor support at home, bad internet connection, and feeding therapy. Forty-three percent of respondents indicated that clients were charged a cancellation rate if the client ended telepractice prematurely or missed a planned session. The amount varied from $20 to half the original rate to the full rate.

Forty-three percent of the respondents were self-employed and 49% were employees of governmental agencies, public, private, and non-profit organizations. Sixty-nine percent of the participants indicated that they were using telepractice for their primary employment. For the respondents who were not using telepractice as part of their primary employment, 59% were self-employed. Schools (i.e., preschool, elementary, and secondary) were the most common facilities for serving clients via telepractice as indicated by 91% of respondents. The second most common was in the client’s home as indicated by 56% of the respondents. Thirteen percent for international and 11% for special day/residential schools were third and fourth, respectively.

Related to Health Insurance Portability and Accountability Act (HIPAA, 1996) compliance, 84% reported using a platform that was promoted as HIPAA compliant, 48% indicated that the client signs a permission form to allow telepractice, 56% had written policies and procedures related to HIPAA, and 58% used HIPAA policies established by the employer. Participants indicated the security measures that were in place to ensure no breaches in confidentiality. Sixty-five percent used unique passwords, 62% used encryption, 60% used a secure connection via virtual private network, 53% used unique meeting numbers, and 50% used hardware/software firewalls.

### PROFILE OF THE PARTICIPANTS BY LICENSING AND LICENSURE REGULATIONS

The respondents reported on the number of state licenses that they maintained: one state (39%), two states (28%), three states (17%), and four or more (15%). Thirty-four percent of respondents reported that they were restricted from doing telepractice due to state licensure regulations, whereas 46% indicated that they were not restricted. One restriction that varies across states may involve providing assessment by telepractice. Eighty-eight percent of participants indicated that the states in which they are licensed allow assessment via telepractice.

### PROFILE OF PARTICIPANTS BY COSTS AND EQUIPMENT

Forty-nine percent of participants indicated that the money needed to begin/implement telepractice was from $500–$2000 and 22% indicated that over $2000 was needed. The participants included the following in the estimate: computer, headphones, microphone, software, marketing materials, telepractice training, licenses in various states, and high speed internet. The large range of costs may be explained by whether or not the respondents were employed by a government agency (49%) or self-employed (43%). Practitioners who were self-employed probably had increased costs to implement telepractice. Fifty-five percent of respondents indicated that costs including training were not reimbursed by an employer. The necessary equipment needed to begin/implement telepractice was determined by a telepractice continuing education course (56%), personal trial and error (45%), internet search (43%), and consulting with a clinician already involved in telepractice (43%). The type of web camera and microphone used varied slightly across client and clinician. According to the respondents, the client was more likely to use the internal microphone (50%) and the internal web camera (63%) on the device. The respondents indicated that the use of internal versus external microphones by clinicians was essentially the same at 41% and 40%, respectively. According to the respondents, clients preferred laptops (59%) over desktops (35%) with only 5% of clients preferring tablets. Clinicians recommended laptops to the clients (57%) and desktops (42%) with no recommendations for tablets or smartphones. In a majority of responses, the equipment needs were supplied by both the clinicians and the clients (46%) and in some cases by the employer (25%). According to the respondents’ judgment, technical difficulties may interfere with telepractice occasionally (53%), rarely (36%), and frequently (8%).

### PROFILE OF PARTICIPANTS BY SYNCHRONOUS AND ASYNCHRONOUS LEARNING

#### OPPORTUNITIES

Synchronous learning opportunities are conducted live and in real-time with both the client and clinician present, whereas asynchronous learning opportunities are completed by the client only outside of the live sessions. Synchronous interactions are typically held through videoconferencing. Respondents indicated the type of platform used for synchronous exchanges: WebEx (42%), Zoom (35%), and other (28%) were most common. The other platforms included VSee, Vidyo, GoToMeeting, custom employer platform, and WiZIQ. Reasons for choosing the various platforms were cost, familiarity, ease of use, encryption, employer-provided, quality, security, reliability, recommendation from the Waldo County General Hospital Speech-Language Pathology Telepractice Training program, and consistency with HIPAA standards. Asynchronous experiences were typically offered through three main forms of delivery: email (73%), recorded videos (38%), and custom programs (20%). The three most common types of asynchronous opportunities involved the following: homework exercises (81%), recording speech samples (31%), and recording communication interaction samples (27%). Clients typically completed the asynchronous activities less than once a week (29%), once a week (20%), once a day (18%), and other times (29%) varied based on client need and involvement of caregiver.

### PROFILE OF PARTICIPANTS BY USE OF CLIENT’S ENVIRONMENT AND CAREGIVER/E-HELPER

Seventy-two percent of respondents indicated using the client’s environment (i.e., the setting in which the client lives, works, and plays) during telepractice sessions. Sixty percent reported using the environment synchronously and 29% reported using the environment asynchronously. For assessment, the client’s environment was never used (33%), rarely used (17%), sometimes used (26%), frequently used (12%), and always used (10%). For treatment, the client’s environment was never used (9%), rarely used (10%), sometimes used (40%), frequently used (17%), and always used (12%). Eighty-nine percent of respondents reported using communicative partners in the client’s environment to utilize telepractice methods. The most common communicative partners used were: caregiver (59%), e-helper (48%), other (30%), children (19%), spouse (17%), and grandparents (15%). Other communicative partners included: parent, teacher, coworkers, instructional aide, and classmates. Based on the judgment of the respondents, 59% indicated that the use of caregivers was used to its full potential in telepractice methods. Forty-five percent reported using caregiver intervention differently for in-person sessions as compared to telepractice sessions, whereas 55% of respondents indicated that caregiver interactions were not used differently. The domains of caregiver practice in telepractice included: assisting with technology (85%), generalization of newly learning behaviors (73%), practice newly learning behaviors (67%), homework (58%), direct intervention (30%), and assist with assessment (26%). Fifty percent of respondents reported that use of caregivers in telepractice was sometimes needed and 29% reported that use of caregivers in telepractice was frequently needed.

### PROFILE OF PARTICIPANTS BY METHOD ADAPTATIONS IN TELEPRACTICE

The length of typical telepractice sessions reported by the respondents varied from 15–30 minutes (26%), 30–45 minutes (42%), 45–60 minutes (28%), and over 60 minutes (3%). Respondents indicated that the session length between in-person and telepractice was essentially equal (80%). The respondents indicated that the following domains needed to be adjusted for effective telepractice sessions: technology (83%), therapy materials (64%), cueing (verbal/visual/physical/tactile, 57%), caregiver/e-helper interaction (55%), environment (46%), reinforcement (39%), SLPs communication (37%), time (26%), and frequency of sessions (8%). Seventy-three percent of respondents indicated that telepractice requires different skills from traditional in-person sessions. The following methods need to be adjusted for telepractice sessions: therapy targets to match technology (69%), motivation (67%), administering standardized tests to a child (63%), reinforcement (61%), cueing (55%), home program/exercises (36%), administering standardized tests to an adult (30%), helping parent administer test to child (13%), and other (5%). Eighty-four percent agreed that additional training was required for telepractice.

### PROFILE OF PARTICIPANTS BY OVERALL IMPRESSIONS OF TELEPRACTICE

Respondents indicated that telepractice improved their ability to work with clients (36% agree and 35% strongly agree). Seventeen percent were neutral as to whether or not telepractice improved their ability to work with clients and only 10% strongly disagreed with the statement. Ninety-six percent of the respondents reported that, in their opinion, clients were satisfied with telepractice, while 92% of the respondents indicated that clinicians were satisfied with therapy delivered via telepractice. The respondents indicated their client’s main complaints about telepractice were using equipment (33%), other (21%), and prefer in-person interaction (14%). Respondents indicated that other complaints included: auditory issues with the microphones, not paid for by insurance/Medicare, and slow internet.

## DISCUSSION

As telepractice models are designed and SLP graduate programs facilitate training in telepractice, some key issues need to be addressed that were highlighted from the results of the current survey. One, either a hybrid approach (i.e., in-person and telepractice) or a telepractice only approach can be used with clients. Deciding which one is best for the client is a major consideration. As we train graduate students in telepractice, we need to help them develop the ability to determine the best approach for each client.

Two, licensure regulations by state need to be targeted in the training of future practitioners; for example, investigating state laws on providing assessment via telepractice is one type of regulation that future practitioners must know when implementing telepractice.

Three, costs of telepractice may extend beyond equipment and include additional training, marketing materials, and maintaining multiple licenses. Clinicians need to consider all that is needed for costs and equipment to be successful with telepractice.

Four, choosing a software program for synchronous exchanges is a major consideration. Clinicians need to uphold HIPAA standards and client confidentiality by creating HIPAA compliant procedures and methods. Future practitioners need to be trained on how to develop such procedures and methods.

Five, as we train future clinicians and design new telepractice models, asynchronous learning opportunities need to be explored and become more prevalent in both in-person and telepractice sessions. Clients and clinicians work together synchronously for a finite amount of time during each week. Asynchronous opportunities extend that finite time to the client’s functional environment. Using caregivers or e-helpers offers an advantage by integrating increased use of asynchronous opportunities.

Six, skills of clinicians need to be developed on how to use the client’s environment and the caregiver or e-helper with activities beyond help with technology. Both the environment and caregiver/e-helper need to be more involved with direct intervention of newly learning skills. The use of the environment in improving client outcomes is supported by the WHO (2001).

Seven, the session length may be equal between in-person and telepractice, but methods must be adapted to be effective in the telepractice environment. Such methods include: communication style and timing, motivation, therapy targets, cueing, reinforcement, etc.

In conclusion, the results of the survey have provided additional information from practicing clinicians using telepractice that extend the work of previous surveys focused on demographics, areas of service delivery, and preparation and training for telepractice (ASHA 2002, 2016; Grogan-Johnson et al., 2015). Such information is needed to design new telepractice models and to facilitate telepractice training in SLP graduate programs.
